# Consumption of ultra‐processed foods could influence the metabolic syndrome odds: A cross‐sectional study

**DOI:** 10.1002/fsn3.3938

**Published:** 2024-01-04

**Authors:** Sanaz Mehrabani, Niloofar Shoaei, Zainab Shateri, Moein Askarpour, Mehran Nouri, Parisa Keshani, Behnam Honarvar, Reza Homayounfar

**Affiliations:** ^1^ Health Policy Research Center, Institute of Health Shiraz University of Medical Sciences Shiraz Iran; ^2^ Department of Clinical Nutrition, School of Nutrition and Food Science Isfahan University of Medical Sciences Isfahan Iran; ^3^ Department of Community Nutrition, School of Nutrition and Food Science, Food Security Research Center Isfahan University of Medical Sciences Isfahan Iran; ^4^ Department of Nutrition and Biochemistry, Faculty of Medicine Ilam University of Medical Sciences Ilam Iran; ^5^ Department of Clinical Nutrition, School of Nutritional Sciences and Dietetics Shiraz University of Medical Sciences Shiraz Iran; ^6^ Students' Research Committee, School of Nutrition and Food Science Shiraz University of Medical Sciences Shiraz Iran; ^7^ Department of Community Nutrition, School of Nutrition and Food Sciences Shiraz University of Medical Sciences Shiraz Iran; ^8^ National Nutrition and Food Technology Research Institute Shahid Beheshti University of Medical Sciences Tehran Iran

**Keywords:** blood pressure, HDL‐C, hyperglycemia, metabolic syndrome, triglycerides, ultra‐processed foods, waist circumference

## Abstract

Metabolic syndrome (MetS) prevalence has augmented globally during recent decades. Over the past years, the consumption of ultra‐processed foods (UPFs) has grown significantly worldwide. So, the present research investigated the association between UPFs and MetS in an Iranian sample. This cross‐sectional research was conducted on people (*n* = 8841) in the Fasa cohort study, Fars province, Iran. The participants' dietary consumption over a year, UPF consumption, and MetS diagnosis were evaluated through a 125‐item modified food frequency questionnaire, the NOVA food group classification, and the Adult Treatment Panel III of the National Cholesterol Education Program, respectively. The association between the quartiles (Q) of UPF intake and the odds of MetS was estimated using the backward LR method of multivariate analysis. In the multivariate model, after adjusting potential confounders, the association between UPF intake and the odds of MetS was significant (Q_4_: odds ratio (OR = 3.27; 95% confidence interval (CI): 2.76–3.89). Also, the odds of increasing triglycerides (TG), blood pressure, and fasting blood sugar (FBS) and decreasing high‐density lipoprotein cholesterol (HDL‐C) were significantly higher in the last quartile compared to the first quartile of UPFs (TG: OR = 1.71; 95% CI: 1.49–1.97, blood pressure: OR = 1.53; 95% CI: 1.30–1.79, FBS: OR = 1.30; 95% CI: 1.10–1.54, and HDL‐C: OR = 1.22; 95% CI: 1.08–1.39). The current research found a relationship between UPF intake and MetS and its components, indicating a diet‐containing UPFs can be related to the occurrence of noncommunicable diseases.

## Graphical abstract

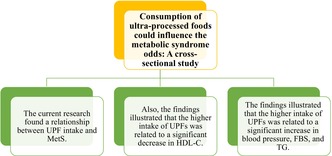

## 1 INTRODUCTION

Metabolic syndrome (MetS) includes a group of metabolic disorders: insulin resistance, hypertension, abdominal adiposity, and dyslipidemia (Fahed et al., 2022). MetS causes a fivefold increment in type 2 diabetes mellitus risk and a twofold increment in cardiovascular disease risk, respectively. This syndrome's prevalence has augmented globally during recent decades, presumably caused by lifestyle, inactivity, and obesity (Canhada et al., 2023).

The exact cause of MetS is unclear, but a complex interaction among environmental, metabolic, and genetic factors may play a role in its occurrence (Feldeisen & Tucker, 2007). Among the modifiable and effective environmental factors on MetS, dietary habits have a special place in the treatment and prevention of MetS (Baxter et al., 2006; Feldeisen & Tucker, 2007). Over the past years, the consumption of ultra‐processed foods (UPFs) has grown significantly globally, replacing healthy food patterns such as nuts, legumes, vegetables, and fruits (Leo & Campos, 2020; Salas‐Salvadó et al., 2011). UPFs typically contain high amounts of saturated and trans‐fatty acids, salt, added sugars, and energy and are poor in fiber, protein, and micronutrients (Martinez‐Perez et al., 2021; Monteiro et al., 2018). Studies have demonstrated that UPFs are related to increased noncommunicable diseases (NCDs), obesity, and overweight (da Costa Louzada et al., 2015; Pagliai et al., 2021).

There is limited evidence of the association between MetS and UPFs. A cohort study of Brazilian adults illustrated that UPF consumption was related to an elevated risk of MetS (Canhada et al., 2023). Also, another study on Brazilian adolescents revealed that the consumption of UPFs was related to the increase in MetS prevalence (Tavares et al., 2012). However, a study on women from Quilombola communities of Alagoas indicated no relationship between UPF consumption and MetS (Barbosa et al., 2023).

Although limited research has investigated the relationship between MetS and UPFs, no study in Iran has assessed this issue. Also, there may be inconsistencies between the different studies' findings due to the differences between the average consumption of UPFs, dietary habits, and lifestyle in each population. So, the present research investigated the association between UPFs and MetS in an Iranian sample.

## 2 MATERIALS AND METHODS

### 2.1 Study participants

This cross‐sectional research was conducted on people living in the village in the Fasa cohort study, Fars province, Iran. Fasa is a small city located in the eastern part of the Fars province. This study is a part of the Prospective Epidemiological Research Studies in Iran (PERSIAN) cohort, which looks at NCDs over a period of time. At first, in every health house in village and small town, healthcare workers (Behvarz) explained the study's aims for participants and invited them to participate in this study based on study protocol (Farjam et al., 2016). The study subjects were 11,097 individuals aged between 35 and 70 residing in the rural region of Sheshdeh, in the suburbs of Fasa City. Additionally, individuals from 24 nearby villages were also included in the study. The permission for this research was issued by the Shiraz University of Medical Sciences Ethics Committee (IR.SUMS.REC.1401.363). Some details of this study have been previously published (Nouri et al., 2023).

**FIGURE 1:**
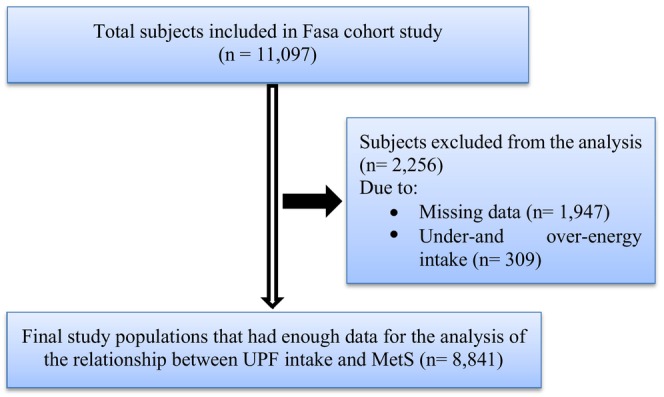
The study flow chart.

Comprehensive data on each person's demographic information, socioeconomic status, physical measurements, dietary habits, and medical background were gathered at the beginning of the study by educated experts (some details of this cohort study have been published (Farjam et al., 2016)). This study assessed the participants' eating habits and food consumption through a modified food frequency questionnaire (FFQ) containing 125 items. The questionnaire was adapted to include Iranian food items. To prevent inaccurate dietary classification, we excluded daily caloric intake below 800 or above 4200 kcal/day. After removing 2256 people because we did not have information on physical activity or due to the over‐ or underestimation of energy intake, 8841 people were included in the study (Figure 1).

### 2.2 Measurements

#### 2.2.1 Baseline characteristics of the study participants

The general information checklist was completed through interviews, including individual information (age (year), gender (male or female), physical activity (MET/day), education (year), current smoking status and alcohol consumption (yes/no), and mediation (yes/no)), home situation, lifestyle status, food habits, and anthropometric data.

In addition, this study used checklists to collect information about the subjects' health issues in terms of NCDs, to find out if they drank alcohol or smoked, and to record the participants' physical activity. Participants were also told to bring their medications to accurately record their medication history during the interview (Farjam et al., 2016). A standard questionnaire was used to calculate the time each participant spent on physical activity (including different types of activities such as work, sleep, walking, exercise, and others). Based on the time spent for each type of activity, metabolic equivalent of task (MET) was measured.

#### 2.2.2 Dietary intake

The participants' eating habits and dietary consumption over a year were assessed through a 125‐item‐modified FFQ (Farjam et al., 2016). At first, based on Iranian home scales, frequency and portion size of each food item were converted to grams, and then Nutritionist IV software was applied to compute nutrient and energy intake (Nutritionist, 1998).

#### 2.2.3 Biochemical assessment

Before the blood samples were drawn from the participants, they fasted for 10–14 h. Then, the samples were kept at −80°C. Frozen plasma samples were used to measure lipid profile and fasting blood sugar (FBS) concentration (Farjam et al., 2016).

#### 2.2.4 Anthropometric and blood pressure assessment

To assess the participants' height and weight, bioelectrical impedance analysis (Tanita BC‐418, Tanita Corp, Japan) was used. In addition, researchers used a nonstretchable tape to measure the size of the hip and waist. Blood pressure was measured twice in both arms, while participants were in a sitting position for 5 min and then again after 15 min by trained nurses. The average blood pressure (mmHg) in both arms was reported as systolic and diastolic blood pressure (Farjam et al., 2016).

#### 2.2.5 Assessment of UPF intake

The NOVA food group classification was utilized to assess UPF consumption. NOVA divides foods into four classes based on how much they have been processed (Petrus et al., 2021). As a result, some beverages and foods consisting of processed meats, salty, and fried snacks, sweetened milk‐based beverages, ice cream, industrial fruit drinks, soft drinks, cakes, biscuits, sweets and pastries, dressings, sauces, margarine, packaged bread, sweets, and buns were selected, and then the energy of each UPFs item was calculated. Finally, to determine the total consumption of UPFs, the average amount consumed per day was divided by the total daily intake of UPFs, and then multiplied by 100.

#### 2.2.6 Assessment of MetS

According to the Adult Treatment Panel (ATP) III of the National Cholesterol Education Program, a person is diagnosed with MetS if they meet three of these criteria: high blood pressure (systolic blood pressure (SBP) >130 mmHg and/or diastolic blood pressure (DBP) >85 mmHg), high FBS (>100 mg/dL), large waist circumference (WC) (>88 cm for women and >102 cm for men), high concentration of triglycerides (TG) (≥150 mg/dL), and low levels of high‐density lipoprotein cholesterol (HDL‐C) (<50 mg/dL for females and <40 mg/dL for males).

#### 2.2.7 Statistical analysis

We applied SPSS (version 26.0) to analyze all the data in this study. If the *p*‐value was less than .05, we considered it statistically significant. The normality of study variables was checked by Kolmogorov–Smirnov test. At first, participants were categorized into quartiles based on energy intake of UPF items and analysis done according to this classification. To compare the baseline characteristics between the groups, we used analysis of variance (ANOVA) and chi‐square tests to analyze continuous and categorical variables, respectively. The association between the quartiles of UPF intake and the odds of MetS, MetS components, and covariates were estimated using the univariate logistic regression. Additionally, the backward LR method of multivariate analysis was applied to consider the effect of confounders (variables with *p*‐value <.25 in univariate test entered in the multivariate analysis).

## 3 RESULTS

According to Table 1, age, gender, physical activity, education years, current smoking status, alcohol history, and medication significantly differed between quartiles of UPF intakes (*p* < .001 for all, except physical activity). Also, UPF, fiber, carbohydrate, and fat intakes were significantly higher, but energy and protein intake in the last quartile of UPFs was lower than the first quartile (*p* < .001 for all). Furthermore, MetS prevalence was 19.9% in the total population and significantly differed between quartiles of food index (*p* < .001).

**TABLE 1:** Baseline characteristics of study participants.

Variables	Ultra‐processed foods (*N* = 8841)					
	Q_1_ (*N* = 2210)	Q_2_ (*N* = 2210)	Q_3_ (*N* = 2211)	Q_4_ (*N* = 2210)	Total	*p*‐value
*Demographic*						
Age (year)	51.13 ± 9.52	49.20 ± 9.48	47.30 ± 9.24	47.10 ± 9.53	48.68 ± 9.58	**<.001**
Gender (%) Male Female	37.4 62.6	40.6 59.4	45.8 54.2	51.9 48.1	43.9 56.1	**<.001**
Physical activity (MET/day)	40.94 ± 10.64	41.00 ± 10.75	41.82 ± 11.37	41.67 ± 12.15	41.36 ± 11.25	**.012**
Education (year)	3.86 ± 3.65	4.55 ± 3.84	5.11 ± 3.96	5.07 ± 3.93	4.65 ± 3.88	**<.001**
Current smoking status, no (%)	85.4	83.4	80.7	75.6	81.3	**<.001**
Alcohol history, no (%)	97.5	96.9	94.8	92.0	95.3	**<.001**
Medication, no (%)	15.2	17.6	21.8	27.3	20.5	**<.001**
*Nutrients*						
UPF intake (kcal/day)	52.10 ± 29.5	133.24 ± 44.48	242.49 ± 79.38	519.94 ± 290.42	236.95 ± 233.70	**<.001**
UPF intake (% energy)	1.87 ± 0.90	4.91 ± 0.95	9.16 ± 1.65	22.78 ± 13.12	9.68 ± 10.39	**<.001**
Energy (kcal/d)	2462.42 ± 644.74	2664.96 ± 700.64	2696.60 ± 733.08	2700.87 ± 835.84	2631.21 ± 738.35	**<.001**
Protein (g/day)	107.88 ± 43.92	96.09 ± 35.84	87.23 ± 33.21	79.57 ± 31.83	92.69 ± 37.99	**<.001**
Carbohydrate (g/day)	420.00 ± 164.67	451.92 ± 167.99	492.76 ± 175.88	558.32 ± 217.19	480.75 ± 189.79	**<.001**
Fat (g/day)	56.61 ± 23.77	63.41 ± 23.58	72.03 ± 25.68	85.53 ± 33.20	69.39 ± 28.93	**<.001**
Fiber (g/day)	26.45 ± 11.13	29.01 ± 11.20	31.87 ± 12.18	36.22 ± 15.72	30.89 ± 13.20	**<.001**
*Metabolic syndrome components*						
Metabolic syndrome (%)	14.4	18.4	20.1	26.6	19.9	**<.001**
SBP (mmHg)	112.39 ± 18.62	111.09 ± 18.41	110.43 ± 17.78	111.47 ± 18.32	111.35 ± 18.29	**.004**
DBP (mmHg)	74.75 ± 11.66	74.36 ± 12.05	74.25 ± 11.79	75.04 ± 11.89	74.60 ± 11.85	.100
TG (mg/dL)	122.96 ± 72.47	129.06 ± 81.29	134.77 ± 88.01	138.17 ± 81.17	131.24 ± 81.12	**<.001**
TC (mg/dL)	184.27 ± 38.72	185.78 ± 39.32	185.53 ± 38.75	185.45 ± 39.76	185.26 ± 39.14	.580
HDL‐C (mg/dL)	51.71 ± 14.55	51.40 ± 16.44	50.54 ± 15.63	50.76 ± 16.99	51.10 ± 15.93	.050
FBS (mg/dL)	94.80 ± 33.05	91.59 ± 28.40	90.95 ± 26.18	93.18 ± 30.17	92.63 ± 29.59	**<.001**
BMI (kg/m^2^)	25.59 ± 4.67	25.67 ± 4.87	25.76 ± 4.97	25.67 ± 4.90	25.67 ± 4.86	.704
WC (cm)	93.26 ± 11.58	93.36 ± 11.87	93.23 ± 11.73	93.06 ± 12.06	93.23 ± 11.81	.864
HC (cm)	99.08 ± 8.77	99.74 ± 8.88	100.01 ± 8.88	99.75 ± 8.79	99.65 ± 8.84	**.004**

*Note*: Values are mean (SD) for continuous and percentage for categorical variables. Using one‐way ANOVA for continuous and chi‐square tests for categorical variables in UPF quartiles.

Abbreviations: BMI, body mass index; DBP, diastolic blood pressure; FBS, fasting blood sugar; HC, hip circumference; HDL‐C, high‐density lipoprotein cholesterol; MET, metabolic equivalent of task; SBP, systolic blood pressure; TC, total cholesterol; TG, triglyceride; UPFs, ultra‐processed foods; WC, waist circumference.

The relationship between study variables and MetS in two univariate and multivariate models in the Fasa cohort's entire population is shown in Table 2. In the univariate model, in the second, third, and last quartiles (Q) of UPFs, the odds of MetS were significantly higher compared to the first (Q_2_: odds ratio (OR) = 1.33; 95% confidence interval (CI): 1.13–1.56—Q_3_: OR = 1.49; 95% CI: 1.27–1.75—Q_4_: OR = 2.14; 95% CI: 1.84–2.49, *p* < .001, for all). Also, in the multivariate model, after adjusting potential confounders, the association between UPF intake and the odds of MetS remained significant (Q_2_: OR = 1.58; 95% CI: 1.32–1.88—Q_3_: OR = 2.03; 95% CI: 1.70–2.42—Q_4_: OR = 3.27; 95% CI: 2.76–3.89, *p* < .001, for all).

**TABLE 2:** Association between study variables and metabolic syndrome in the total population of Fasa cohort.

Variables	Metabolic syndrome			
	Total population (*N* = 8838)			
	Univariate OR (95% CI)	*p*‐value	Multivariate OR (95% CI)	*p*‐value
UPFs				
UPF Q_1_	Ref.	–	Ref.	–
UPF Q_2_	**1.33 (1.13–1.56)**	**<.001**	**1.58 (1.32–1.88)**	**<.001**
UPF Q_3_	**1.49 (1.27–1.75)**	**<.001**	**2.03 (1.70–2.42)**	**<.001**
UPF Q_4_	**2.14 (1.84–2.49)**	**<.001**	**3.27 (2.76–3.89)**	**<.001**
Demography				
Age (year)	**1.03 (1.03–1.04)**	**<.001**	**1.04 (1.03–1.05)**	**<.001**
Energy (kcal)	1.00 (1.00–1.00)	.267	**–**	–
Education (year)	**0.89 (0.88–0.91)**	**<.001**	**0.94 (0.98–1.00)**	**<.001**
Physical activity (MET min/week)	**0.96 (0.96–0.97)**	**<.001**	**0.98 (0.97–0.98)**	**<.001**
BMI (kg/m^2^)	**1.20 (1.19–1.22)**	**<.001**	**1.19 (1.18–1.21)**	**<.001**
Medication				
No	Ref.	–	Ref.	–
Yes	**0.29 (0.24–0.35)**	**<.001**	**0.57 (0.46–0.70)**	**<.001**
Smoke exposure				
No	Ref.	–	Ref.	–
Yes	**0.29 (0.24–0.35)**	**<.001**	**0.60 (0.48–0.75)**	**<.001**

*Note*: Significant values are shown in bold. Missing values in each variable were excluded from the analyses. Using backward LR method for multivariate analysis.

Abbreviations: BMI, body mass index; CI, confidence interval; MET, metabolic equivalent of task; OR, odds ratio; UPFs, ultra‐processed foods.

The relationship between UPFs and MetS components in univariate is reported in Table 3. According to Table 3, the odds of increasing WC and FBS among the quartiles of UPFs were significantly lower (WC: Q_3_: OR = 0.87; 95% CI: 0.78–0.99—Q_4_: OR = 0.87; 95% CI: 0.77–0.98; and FBS: Q_2_: OR = 0.74; 95% CI: 0.62–0.87—Q_3_: OR = 0.71; 95% CI: 0.60–0.84). Still, the association between UPF intake and the odds of low HDL‐C and high TG were significantly higher in the second, third, and last quartiles compared to the first one (TG: Q_2_: OR = 1.18; 95% CI: 1.03–1.36—Q_3_: OR = 1.39; 95% CI: 1.21–1.59—Q_4_: OR = 1.59; 95% CI: 1.39–1.82; and HDL‐C: Q_2_: OR = 1.23; 95% CI: 1.09–1.39—Q_3_: OR = 1.25; 95% CI: 1.11–1.41—Q_4_: OR = 1.28; 95% CI: 1.13–1.44).

**TABLE 3:** Association between study variables and metabolic syndrome components in the total population of Fasa cohort in univariate analysis.

Metabolic syndrome components	High waist circumference		High triglyceride		Low HDL‐C		High fasting blood sugar		High blood pressure	
Variables	Univariate OR (95% CI)	*p*‐value	Univariate OR (95% CI)	*p*‐value	Univariate OR (95% CI)	*p*‐value	Univariate OR (95% CI)	*p*‐value	Univariate OR (95% CI)	*p*‐value
UPFs										
UPFs Q_1_	Ref.	–	Ref.	–	Ref.	–	Ref.	–	Ref.	–
UPFs Q_2_	1.01 (0.90–1.14)	.822	**1.18 (1.03–1.36)**	**.014**	**1.23 (1.09–1.39)**	**.001**	**0.74 (0.62–0.87)**	**<.001**	1.08 (0.93–1.26)	.266
UPFs Q_3_	**0.87 (0.78–0.99)**	**.033**	**1.39 (1.21–1.59)**	**<.001**	**1.25 (1.11–1.41)**	**<.001**	**0.71 (0.60–0.84)**	**<.001**	0.96 (0.82–1.12)	.634
UPFs Q_4_	**0.87 (0.77–0.98)**	**.024**	**1.59 (1.39–1.82)**	**<.001**	**1.28 (1.13–1.44)**	**<.001**	0.97 (0.83–1.14)	.793	1.15 (0.99–1.33)	.061
Demography										
Age (year)	**1.01 (1.00–1.01)**	**<.001**	**1.00 (1.00–1.01)**	**.005**	**0.98 (0.98–0.99)**	**<.001**	**1.06 (1.06–1.07)**	**<.001**	**1.06 (1.05–1.06)**	**<.001**
Energy (kcal)	**1.00 (1.00–1.00)**	**.014**	1.00 (1.00–1.00)	.076	1.00 (1.00–1.00)	.793	**1.00 (1.00–1.00)**	**.001**	1.00 (1.00–1.00)	.331
Education (year)	**0.90 (0.89–0.91)**	**<.001**	1.00 (0.98–1.01)	.917	0.99 (0.98–1.01)	.885	**0.88 (0.87–0.90)**	**<.001**	**0.91 (0.90–0.93)**	**<.001**
Physical activity (MET‐min/week)	**0.95 (0.94–0.95)**	**<.001**	**0.98 (0.98–0.99)**	**<.001**	**0.98 (0.97–0.98)**	**<.001**	**0.97 (0.96–0.98)**	**<.001**	**0.98 (0.98–0.99)**	**<.001**
BMI (kg/m^2^)	**1.56 (1.53–1.59)**	**<.001**	**1.09 (1.08–1.10)**	**<.001**	**1.06 (1.05–1.07)**	**<.001**	**1.08 (1.06–1.09)**	**<.001**	**1.09 (1.08–1.10)**	**<.001**
Medication										
No	Ref.	–	Ref.	–	Ref.	–	Ref.	–	Ref.	–
Yes	**0.08 (0.07–0.10)**	**<.001**	0.96 (0.86–1.08)	.579	**0.76 (0.69–0.85)**	**<.001**	**0.62 (0.53–0.73)**	**<.001**	**0.62 (0.53–0.72)**	**<.001**
Smoke exposure										
No	Ref.	–	Ref.	–	Ref.	–	Ref.	–	Ref.	–
Yes	**0.09 (0.08–0.11)**	**<.001**	0.89 (0.79–1.00)	.068	0.92 (0.83–1.03)	.176	**0.53 (0.44–0.53)**	**<.001**	**0.51 (0.43–0.60)**	**<.001**

*Note*: Significant values are shown in bold. Missing values in each variable were excluded from the analyses. Using backward LR method for multivariate analysis.

Abbreviations: BMI, body mass index; CI, confidence interval; HDL‐C, high‐density lipoprotein cholesterol; MET, metabolic equivalent of task; OR, odds ratio; UPFs, ultra‐processed foods.

The relationship between UPFs and MetS components in multivariate is shown in Table 4. As shown in Table 4, compared to the first quartile of UPFs, the odds of increasing TG and blood pressure and decreasing HDL‐C were significantly higher in the second, third, and last quartiles (TG: Q_2_: OR = 1.21; 95% CI: 1.05–1.39—Q_3_: OR = 1.45; 95% CI: 1.26–1.66—Q_4_: OR = 1.71; 95% CI: 1.49–1.97, HDL‐C: Q_2_: OR = 1.20; 95% CI: 1.06–1.36—Q_3_: OR = 1.21; 95% CI: 1.07–1.37—Q_4_: OR = 1.22; 95% CI: 1.08–1.39, and blood pressure: Q_2_: OR = 1.25; 95% CI: 1.07–1.47—Q_3_: OR = 1.24; 95% CI: 1.05–1.46—Q_4_: OR = 1.53; 95% CI: 1.30–1.79). Also, the odds of increased FBS were significantly higher in the last quartile of UPF than in the first (Q_4_: OR = 1.30; 95% CI: 1.10–1.54).

**TABLE 4:** Association between study variables and metabolic syndrome components in the total population of Fasa cohort in multivariate analysis.

Metabolic syndrome components	High waist circumference		High triglyceride		Low HDL‐C		High fasting blood sugar		High blood pressure	
Variables	Multivariate OR (95% CI)	*p*‐value	Multivariate OR (95% CI)	*p*‐value	Multivariate OR (95% CI)	*p*‐value	Multivariate OR (95% CI)	*p*‐value	Multivariate OR (95% CI)	*p*‐value
UPFs										
UPF Q_1_	Ref.	–	Ref.	–	Ref.	–	Ref.	–	Ref.	–
UPF Q_2_	–	–	**1.21 (1.05–1.39)**	**.008**	**1.20 (1.06–1.36)**	**.003**	0.84 (0.70–1.00)	.050	**1.25 (1.07–1.47)**	**.005**
UPF Q_3_	–	–	**1.45 (1.26–1.66)**	**<.001**	**1.21 (1.07–1.37)**	**.002**	0.92 (0.77–1.10)	.400	**1.24 (1.05–1.46)**	**.009**
UPF Q_4_	–	–	**1.71 (1.49–1.97)**	**<.001**	**1.22 (1.08–1.39)**	**.001**	**1.30 (1.10–1.54)**	**.002**	**1.53 (1.30–1.79)**	**<.001**
Demography										
Age (year)	**0.98 (0.97–0.99)**	**<.001**	**1.01 (1.00–1.01)**	**<.001**	**0.98 (0.98–0.99)**	**<.001**	**1.06 (1.05–1.07)**	**<.001**	**1.06 (1.06–1.07)**	**<.001**
Energy (kcal)	–	–	**1.00 (1.00–1.00)**	**.003**	–	–				
Education (year)	**0.84 (0.82–0.85)**	**<.001**	–	–	–	–	0.98 (0.96–1.00)	.070		
Physical activity (MET min/week)	**0.95 (0.94–0.96)**	**<.001**	**0.99 (0.99–1.00)**	**.037**	**0.98 (0.97–0.98)**	**<.001**	**0.98 (0.97–0.99)**	**<.001**		
BMI (kg/m^2^)	**1.63 (1.59–1.67)**	**<.001**	**1.10 (1.08–1.11)**	**<.001**	**1.06 (1.05–1.07)**	**<.001**	**1.08 (1.06–1.09)**	**<.001**	**1.09 (1.08–1.11)**	**<.001**
Medication										
No	Ref.	–	Ref.	–	Ref.	–	Ref.	–	Ref.	–
Yes	**0.12 (0.10–0.16)**	**<.001**	–	–	**0.81 (0.71–0.92)**	**.002**	–	–		
Smoke exposure										
No	Ref.	–	Ref.	–	Ref.	–	Ref.	–	Ref.	–
Yes	**0.24 (0.19–0.30)**	**<.001**	**1.19 (1.05–1.36)**	**.007**	**1.33 (1.16–1.52)**	**<.001**	**0.72 (0.60–0.88)**	**.001**	**0.68 (0.57–0.81)**	**<.001**

*Note*: Significant values are shown in bold. Missing values in each variable were excluded from the analyses. Using backward LR method for multivariate analysis.

Abbreviations: BMI, body mass index; CI, confidence interval; HDL‐C, high‐density lipoprotein cholesterol; MET, metabolic equivalent of task; OR, odds ratio; UPFs, ultra‐processed foods.

## 4 DISCUSSION

This cross‐sectional study's findings from the Fasa cohort study suggested that higher UPF intake was related to a higher MetS prevalence. Also, the findings illustrated that the higher intake of UPFs was related to a significant decrease in HDL‐C and an increase in blood pressure, FBS, and TG.

The present research revealed that MetS prevalence was 26.6% in the highest UPF quartile. We also observed a positive relationship between MetS and UPF intake. Our study's findings are in line with previous studies. A cross‐sectional study on Canadian adults indicated a positive and significant association between MetS and UPF consumption (Lavigne‐Robichaud et al., 2018). A cross‐sectional study by Tavares et al. on Brazilian adolescents also showed that high intake of UPF is related to higher odds of MetS (Tavares et al., 2012). In addition, another cross‐sectional study of US adults found that MetS prevalence increased by 4% with a 10% increase in UPF intake (da Costa Louzada et al., 2015). However, a cohort study of Brazilian adults revealed no relationship between UPF and MetS risk (Magalhães et al., 2022). The difference in research sample and design can justify the difference in the mentioned study with previous studies and the present findings.

One of the effect mechanisms of UPFs on the occurrence of MetS is due to the nutrients it contain. Studies have indicated that UPFs contain high amounts of sodium, free/added sugars, and saturated fat and are poor in micronutrients and fiber (Steele et al., 2019). It has been shown that added sugars and saturated fatty acids are associated with increased risk of MetS (Martínez‐González & Martín‐Calvo, 2013), while high consumption of fish, whole grains, fruits, vegetables, polyunsaturated fatty acids, monounsaturated fatty acids, and fiber is associated with a reduced risk of MetS (Calton et al., 2014; Martínez‐González & Martín‐Calvo, 2013; Pérez‐Martínez et al., 2017). Since satiety plays an important role in dietary intake, UPFs are enjoyable to consume due to their high sugar and salt content. As a result, it reduces the satiety potential, increases intake (Fardet & Rock, 2019), and causes obesity. Obesity can be related to an increased MetS risk (Palmer & Toth, 2019). It has also been suggested that UPF consumption may affect health and metabolism by modulating the function and composition of gut microbiota (Cuevas‐Sierra et al., 2019; Muralidharan et al., 2021). The environment created in the gut by UPFs is a trigger for oxidative stress and systemic low‐grade inflammatory changes (Leo & Campos, 2020). The inflammation and oxidative stress role in MetS has been established (Silveira Rossi et al., 2022). Furthermore, UPF is related to less intake of nuts, fish, legumes, vegetables, and fruits (Martínez Steele et al., 2017; Steele et al., 2019), which have been demonstrated to decrease MetS risk (Martínez‐González & Martín‐Calvo, 2013).

According to the result of the present study, a negative association was seen between age and high WC. Previous studies have indicated that WC tends to increase as individuals age (Stevens et al., 2010; Zamboni et al., 2003). It is possible that the negative association we noted in our study is a result of the dietary lifestyle changes observed with increasing age, particularly the adoption of healthier diets. As demonstrated in Table 1, individuals in the highest quartile of UPF consumption were younger than those in the lowest quartile. Additionally, the result indicated a negative correlation between age and low HDL‐c. In line with our result, a previous study revealed that aging is positively associated with increased HDL‐c levels after controlling for factors including TG level (Harman et al., 2011). However, some studies revealed HDL‐c levels decreased with aging (Cho et al., 2019; Wilson et al., 1994). The negative association between age and low HDL‐C in the current study could be attributed to the fact that elderly subjects tend to consume healthier diets due to the diseases that are more common among older individuals.

Additionally, our study has found that there is a negative link between education level and high WC. Previous studies have also indicated similar results to present study (Hermann et al., 2011). This implies that not having enough education can have a significant impact on obesity‐related behavior, such as choosing diet and conducting physical activity.

Additionally, a negative association between physical activity and high WC, high TG, low HDL‐c level, and high FBS concentration. In accordance with the results of the current study, a recent meta‐analysis revealed that regular aerobic exercise leads to decrease in waist circumference (Armstrong et al., 2022). Additionally, other studies revealed that regular leisure‐time physical activity is a tool in the fight against obesity in older adults. This research revealed that those who engage in leisure‐time physical activity are less likely to develop both general and abdominal obesity, as well as they have lower BMIs and WC measurements (Cárdenas Fuentes et al., 2018). Exercise significantly enhances the activity of hormone‐sensitive lipases (HSLs), resulting in a more efficient conversion of TG to free fatty acids (Enevoldsen et al., 2001; Haemmerle et al., 2002; Ogasawara et al., 2010). Also, exercise can increase the secretion of a protein hormone called adiponectin (Becic et al., 2018), which is mainly produced by adipocytes and helps regulate glucose levels and fatty acid breakdown. It is a well‐established fact that adiponectin boosts the levels of ATP‐binding cassette transporter A1 and lipoprotein lipase (LPL), while also lowering the levels of hepatic lipase (Yanai & Yoshida, 2019).

Furthermore, the present study revealed a negative correlation between medication usage and high WC as well as low HDL‐c levels. This could be attributed to the fact that individuals who take medication may alter their diet and consume healthier foods, or it could be due to the impact of medications on their weight and lipid profile.

Also, the current study demonstrated that there was a negative association between smoke exposure and high WC, high FBS, and high blood pressure, while this association was positive between smoke exposure and high TG and low HDL‐C. In contrast with our result, previous studies revealed that smoking was related to unfavorable glycemic control due to increased insulin resistance (Chiolero et al., 2008; Sargeant et al., 2001; Sia et al., 2022). However, our analysis showed that the number of smokers consuming UPFs in Q4 was lower than in Q1, which may have affected the result of our study. In addition, in line with this result, a meta‐analysis demonstrated that in observational analyses, current smoking was associated with lower blood pressure and lower hypertension risk, but with a higher resting heart rate, compared to never smoking (Linneberg et al., 2015). However, some studies found that blood pressure increased in passive smoking exposure (Mahmud & Feely, 2004; Seki et al., 2010). It is important to note that self‐reported tobacco use is highly susceptible to misclassification and reporting bias, and does not account for differences in smoking topography including the number of smoking used by subjects (Munafò et al., 2012). However, current study showed a positive association between smoke exposure and high TG levels. In line with our result, data from recent meta‐analysis clearly showed that smokers had TG levels that were 0.50 mmol/L higher than nonsmokers (van der Plas et al., 2023). Also, exposure to smoke may increase insulin resistance by activating mammalian targets of rapamycin, which in turn can lead to increased FBS levels (Bergman et al., 2012).

Our findings also illustrated that the intake of UPFs was associated with the components of MetS, such as high blood pressure, FBS, TG, and low HDL‐C. In a cohort study of middle‐aged Spanish people, UPF consumption was shown to be related to a high risk of hypertension (Mendonça et al., 2017). A systematic review and meta‐analysis study also revealed that high consumption of UPFs was related to a high risk of hypertension in adults (Wang et al., 2022). Moreover, in cross‐sectional research on Brazilian schoolchildren, UPF consumption was demonstrated to be related to increasing fasting glucose (Rinaldi et al., 2016). In addition, in previous studies, the association between UPF intake and hypertriglyceridemia and reduction in HDL‐C has been shown (da Silva Scaranni et al., 2021, 2023; Donat‐Vargas et al., 2021). Excess energy intake from UPF consumption causes obesity, which can certainly be a cause of cardiometabolic risk factors (Mambrini et al., 2023). Many UPFs contain high amounts of sodium, a known risk factor for hypertension (Filippini et al., 2022). Also, added fructose contributes to oxidative stress and low‐grade inflammation and causes damage to beta cells, resulting in decreased insulin secretion. Moreover, added sugar causes insulin resistance in the whole body and the liver (Lustig, 2010). UPFs also contain saturated and trans‐fatty acids, which can increase dyslipidemia risk (Mambrini et al., 2023). Studies have shown the impact of trans‐fatty acids on dyslipidemia (Matthan et al., 2004; Mensink et al., 2003).

Our study has several strengths. This study is the first to examine UPF consumption and MetS in the Iranian population. Also, the current study's results were concluded from a large sample size. Moreover, the relationship between UPFs and components of MetS was assessed. Furthermore, considering many confounding factors in the statistical analysis was one of the other strengths of the current study. One of the limitations of the research is its cross‐sectional nature, which made it impossible to derive a causal relationship. Also, there may be other confounding factors that have not been investigated in the research. Moreover, using FFQ, which may have recall bias, is another current study limitation.

## 5 CONCLUSIONS

The current cross‐sectional research found a relationship between UPF intake and MetS and its components in a large sample of Iran's population. These findings show that a diet containing UPFs can be related to the occurrence of NCDs. More studies are needed to confirm the present results.

## AUTHOR CONTRIBUTIONS

Sanaz Mehrabani, Niloofar Shoaei, Zainab Shateri, Moein Askarpour, Mehran Nouri, and Parisa Keshani contributed to writing the first draft. Mehran Nouri and Behnam Honarvar contributed to all data and statistical analysis and interpretation of data. Behnam Honarvar and Reza Homayounfar contributed to the research concept, supervised the work, and revised the manuscript. All authors read and approved the final manuscript.

## CONFLICT OF INTEREST STATEMENT

None.

## ETHICS STATEMENT AND CONSENT TO PARTICIPATE

This study was conducted in accordance with the ethical standards of the Declaration of Helsinki and was approved by the Medical Research and Ethics Committee of the Shiraz University of Medical Science (IR.SUMS.REC.1401.363). All participants read and signed the informed consent form.

## Data Availability

The datasets used and/or analyzed during the current study are available from the corresponding author upon reasonable request.
